# The Roles of Lipoprotein in Psoriasis

**DOI:** 10.3390/ijms21030859

**Published:** 2020-01-29

**Authors:** Chun-Ming Shih, Chang-Cyuan Chen, Chen-Kuo Chu, Kuo-Hsien Wang, Chun-Yao Huang, Ai-Wei Lee

**Affiliations:** 1 Department of Internal Medicine, School of Medicine, College of Medicine, Taipei Medical University, Taipei 11031, Taiwan; cmshih53@tmu.edu.tw (C.-M.S.); cyhuang@tmu.edu.tw (C.-Y.H.); 2 Cardiovascular Research Center, Taipei Medical University Hospital, Taipei 11031, Taiwan; 3 Taipei Heart Institute, Taipei Medical University, Taipei 11031, Taiwan; 4 Department of Anatomy and Cell Biology, School of Medicine, College of Medicine, Taipei Medical University, Taipei 11031, Taiwan; chance03070307@gmail.com; 5 Department of Emergency Medicine, Taichung Veterans General Hospital, Taichung 40705, Taiwan; drckchu@gmail.com; 6 Department of Dermatology, Taipei Medical University Hospital, Taipei 11031, Taiwan; khwang40@gmail.com

**Keywords:** lipid, psoriasis, inflammation

## Abstract

The association between psoriasis and cardiovascular disease risk has been supported by recent epidemiological data. Patients with psoriasis have an increased adjusted relative risk for myocardial infarction. As such, the cardiovascular risk conferred by severe psoriasis may be comparable to what is seen with other well-established risk factors, such as diabetes mellitus. Previous studies demonstrated that low-density lipoprotein (LDL) plays critical roles during atherogenesis. It may be caused by the accumulation of macrophages and lipoprotein in the vessel wall. Oxidized LDL (ox-LDL) stimulates the expression of adhesion molecules, such as ICAM-1 and VCAM-1, on endothelial cells and increases the attachment of mononuclear cells and the endothelium. Even though previous evidence demonstrated that psoriasis patients have tortuous and dilated blood vessels in the dermis, which results in the leakage of ox-LDL, the leaked ox-LDL may increase the expression of adhesion molecules and cytokines, and disturb the static balance of osmosis. Therefore, exploration of the relationship between hyperlipidemia and psoriasis may be another novel treatment option for psoriasis and may represent the most promising strategy.

## 1. Introduction

Psoriasis is a chronic inflammatory disease related to many diseases, especially cardiovascular disease. Among these diseases, atherosclerosis plays the most important role [1]. Atherosclerosis is caused by inflammation and an imbalance of the lipid metabolism. Psoriasis and atherosclerosis not only share the same cytokines involved in the immunological mechanism, such as interleukin (IL)-17, but also have common angiogenic factors and oxidative pathways [2]. In addition, both of them have similar lipid profiles, including decreased high-density lipoprotein (HDL) levels and/or increased low-density lipoprotein (LDL) levels [3]. In the pathological process of atherosclerosis, the accumulation of cholesterol triggers the production of pro-inflammatory cytokines, such as tumor necrosis factor alpha (TNFα), and also leads to the aggregation of monocytes and differentiation into foam cells [4]. TNFα eventually induces an inflammatory cascade in blood vessels [5]. In chronic inflammation, TNFα may also influence the lipid profile, such as LDL-C levels, via a decreased concentration of apolipoproteins. Moreover, TNFα lowers the quality of lipoprotein by inducing the production of LDL and oxLDL and reducing the level of HDL-C at the same time [6]. Oxidized LDL (oxLDL) not only exacerbates inflammation but also promotes cholesterol accumulation in lysosomes, which eventually leads to cell death [7]. On the other hand, HDL has a reverse cholesterol transport (RCT) function, anti-oxidative capacity, and anti-inflammatory properties by regulating dendritic cells’ (DCs) differentiation [8], and reducing T cell activation and IL-12 production [9]. However, these properties are reduced during chronic inflammation, such as psoriasis [10]. Previous studies have clarified the immunological pathway of psoriasis; however, the mechanism between psoriasis and an abnormal lipid profile remains unknown. Thus, identification of the relationship between hyperlipidemia and psoriasis is of paramount importance to develop a new therapeutic prospect for psoriasis.

## 2. Psoriasis

### 2.1. The Etiology of Psoriasis

Psoriasis is a chronic inflammatory skin disease related to immune inheritance. However, to date, the true cause of the disease remains unclear. According to epidemiological statistics, approximately 1%–3% of people worldwide develop psoriasis every year [11]. Psoriasis has long been considered a skin disease. However, according to recent research findings, psoriasis is actually a multisystem disease. It may be related to the occurrence and course of other diseases, including rheumatological (psoriatic arthritis (PsA)), cardiovascular and psychiatric complications [12,13], as well as cardiometabolic diseases, such as obesity, hypertension, and dyslipidemia [14]. At present, comorbid cardiovascular diseases are the main cause of death in patients with psoriasis [15]. The risk of suffering myocardial infarction in patients with severe psoriasis is seven times that in individuals with corresponding age, sex, body mass index (BMI), and cardiovascular risk factors, and the risk of cardiovascular mortality increases by 57% in patients with severe psoriasis [16]. In addition, patients with psoriasis are at a higher risk for cardiovascular diseases. Psoriasis is also related to accelerated atherosclerosis. It has been found that all T cells involved in the pathogenesis of psoriasis are also involved in atherosclerosis [17]. Clinically, psoriasis is divided into many categories, and psoriasis vulgaris (PV) accounts for approximately 90%. Psoriasis often causes symptoms, such as desquamation, skin redness, and itching. In addition to affecting appearance, psoriasis causes great psychological pressure and social distress to patients, thus reducing quality of life [18].

### 2.2. The Molecular Mechanism of Psoriasis

Previous studies have shown that CD4+ helper T cells may differentiate into regulatory T cells and effector T cells (including T-helper cell type 1 (Th1) cells, T-helper cell type 2 (Th2) cells, T-helper cell type 17 (Th17) cells, follicular helper T (Tfh) cells, and regulatory T cells (Tregs)), which are subsequently activated. Before the discovery of other cell lineages, Th1 and Th2 cells were considered to be the only T cells that differentiated from progenitor CD4+ helper T cells [19]. However, a new CD4+ helper T cell subset was discovered, named Th17 cells [20]. Th17 cells produce interleukin 17 (IL-17). Among all Th cell subsets, IL-17-producing T cells play an important role in autoimmune diseases, including multiple sclerosis, psoriasis, inflammatory bowel diseases, and steroid-resistant asthma [21]. At present, psoriasis is generally considered to be derived from the chronic activation of the IL-23/Th17 cell signaling axis, in which dominant T cell subsets are Th1 and Th17 cells. The cytokines released by Th1 and Th17 cells facilitate immune cell aggregation, promote keratinocyte proliferation, and increase the inflammatory response [22]. More precisely, CD4+ and CD8+ T cells with an IL-17-secretory phenotype are most important because they produce pro-inflammatory cytokines IL-17, IL-22, and tumor necrosis factor alpha (TNFα) [23]. The pathogenic mechanism of psoriasis is divided into the primary initiation phase and the chronic inflammatory phase. In the primary initiation phase, the pathogenic inflammatory response is initiated. The subsequent chronic inflammatory phase is continued by feedback loops and amplification signals. During the development of psoriasis, T cell predominance shifts from Th1 predominance in the initiation phase to Th17 predominance in the chronic inflammatory phase [24]. External stimuli, such as trauma, infection, or drug administration, cause the release of self-nucleotides. Self-nucleotides then bind to antimicrobial peptides (AMPs) released by keratinocytes, forming complexes [19]. AMPs are positively charged proteins and members of the innate immune system and include cathelicidin antimicrobial peptide (CAMP), pro-inflammatory cytokines and chemokines (for example, TNF, IL-17, IL-22, and CC-chemokine ligand 20 (CCL20), among numerous others), and angiogenic factors [24]. Among the AMPs, CAMP is not produced when keratinocytes are healthy. However, CAMP is upregulated when epidermal cells are destroyed. In psoriasis lesions, the increase in CAMP production initiates pathogenic interferon (IFN) signaling cascades and the activation of DCs, resulting in uncontrolled inflammatory reactions [25]. The complexes of self-nucleotides and AMPs then bind to the receptors on antigen-presenting cells, such as toll-like receptor 7 (TLR7) and TLR9 on the surface of plasmacytoid dendritic cells (pDCs). pDCs are a member of the innate immune system circulating in the blood. After antigen presentation, pDCs promote the activation and clonal expansion of antigen-specific CD8+ T cells, and such processes occur in the dermis (activation of memory resident T cells) and local lymph nodes (activation of naive T cells). In addition, in the initiation phase of psoriasis, pDCs release the inflammatory mediators IFNα and IFNβ, thereby stimulating the secretion of pro-inflammatory mediators (such as IL-12, IL-23, and TNF) by myeloid DCs. Subsequently, the activated CD8+ T cells migrate to the epidermal layer and encounter class I major histocompatibility complex (MHC) receptors on the surface of keratinocytes (or melanocytes), triggering the local release of soluble factors, including cytokines, chemokines, and innate immune mediators, to increase local inflammation and stimulate keratinocyte proliferation [24]. In addition to being a skin barrier, keratinocytes release inflammatory cytokines, such as TNF; express IL-17 receptors; and participate in the initiation and amplification of psoriasis [26]. The IL-17 family consists of 6 members, IL-17A to IL-17F, among which IL-17A is the most studied member while IL-17E (also known as IL-25) is an inhibitor of IL-17 [27]. In addition to IL-17A, Th17 cells also release various inflammatory cytokines, including IL-17F, IL-21, IL-22, and granulocyte macrophage colony-stimulating factor (GM-CSF). Therefore, targeting the Th17 cell subset is more therapeutically effective than targeting a single cytokine [21]. Innate immunity mediators stimulate the activation of T cell populations, such as Th1, Th17, and Th22 cells, and then release additional cytokines and chemokines. In particular, IL-1 allows Th17 cells to respond to IL-23 [24]. Th17 cells then release IL-17, IL-22, TNF-α, and other cytokines [28], and participate in the macrophage-dependent and -independent stimulation of DCs, thereby enhancing the immune response [29]. They may participate in the production of angiogenic inflammatory mediators, including monocyte chemoattractant protein (MCP-1), nitric oxide, and vascular endothelial growth factor [30]. The expression of vascular endothelial growth factor receptors on epidermal cells induces cardiovascular endothelial hyperplasia and the expression of adhesion molecules in the vascular endothelium, thereby aggregating immune cells. Angiogenic factors induce tortuous characteristics in papillary dermal vessels at the sites of psoriasis lesions, manifesting as the Auspitz sign. In addition, IL-17 acts on the IL-17 receptor on keratinocytes to stimulate the production of TNF and CCL20 (a chemotactic for T cells and DCs208) by keratinocytes [24]. IL-17 and TNF and/or other pro-inflammatory cytokines stimulate the activation of defensins and chemokines to promote host defense and the accumulation of other immune cells [31]. IL-22 is related to the pathological characteristics of psoriasis, including epidermal hyperplasia, acanthosis, and parakeratosis. Important transcription factors in psoriasis include cyclic AMP, the Janus kinase (JAK) signal transducer and activator of transcription (STAT) family, and nuclear factor-κB (NF-κB). Activation of these transcription factors results in the production of other factors, such as TNF and IL-17, as well as downstream amplification loops. Notably, although IL-17 is a signature cytokine of TH17 cells, other innate and acquired immune cells also produce IL-17, including CD8+ T cells, γδ T cells, innate lymphoid cells, and natural killer (NK) T cells [32]. Compared with a healthy control group, patients with psoriasis display higher serum concentrations of IL-17. However, the paradigm of Th17 cells as the main cellular source of IL-17 is no longer fully valid [33]. Recent studies have noted that the innate immune system, including neutrophils, mast cells, γδ T cells, and innate lymphoid cells, is the most important source of IL-17 in patients with psoriasis [34].

## 3. The Relationships among Psoriasis, Cardiovascular Diseases, and Dyslipidemia

### 3.1. Normal Function of Fat and Cholesterol in Skin Cells

The stratum corneum is the outermost layer of the epidermis, and is a barrier protecting humans from the external environment. In addition to protecting humans against external harm, the stratum corneum prevents water loss from the body [35]. The structure of the stratum corneum is often described as bricks and mortar [36]. The bricks are fully differentiated keratinocytes, mostly consisting of keratin filaments and filaggrin [37]. The mortar refers to intracellular lipids, which consist of ceramides (CERs), free fatty acids (FFAs), and cholesterol. These lipids are arranged into a lamellar structure, and CERs account for 50%. CERs are the main polar lipids in the stratum corneum, and their basic composition is a sphingoid base connected to fatty acids by amide bonds. However, the composition of CERs varies appreciably in the human body. CERs are divided into 12 categories according to the head group composition or fatty acid esterification [38]. CERs play an important role in skin barriers, cell adhesion, and epidermal differentiation. In addition, CERs act as second messengers in stress-induced apoptosis [39]. CERs induce the apoptosis of tumor cells and reduce the resistance of tumor cells to chemotherapy drugs [40]. In psoriasis, the barrier function of the skin and water loss by the epidermis are mainly related to an abnormal composition ratio of CERs [41]. However, the total amount of CERs does not differ significantly between patients with psoriasis and healthy individuals [42]. Studies have shown that prosaposin, a precursor of saposin, and its mRNA were decreased in patients with psoriasis [43]. Saposins are a class of nonenzymatic proteins involved in the hydrolysis of sphingolipids, including the postsecretory glucosyl-CERs in the stratum corneum [44]. Sphingomyelinase is also significantly downregulated in the stratum corneum of patients with psoriasis. The reduction in the enzymes involved in CER production and metabolism may lead to decreases in ceramide 1 and other CERs at the sites of psoriasis lesions [41] as well as decreases in long-chain CERs containing ester-linked fatty acids and CERs containing phytosphingosine, thereby altering CER composition and increasing transepidermal water loss. In addition, the proportion of the three major intracellular lipids varies in the stratum corneum. At the sites of psoriasis lesions, the FFA content is significantly reduced while the cholesterol content is slightly increased [42]. Cholesterol constitutes approximately 25% of animal cell membranes and maintains cell integrity and fluidity. Moreover, the dynamic arrangement of cholesterol enhances the coating ability of the cell membrane, allowing the cell membrane to increase fluidity at low temperatures and increase stability at high temperatures [45]. For circulating low-density lipoprotein (LDL), cholesterol is transported via LDL receptor (LDL-R)-mediated endocytosis of holo-particles. Cholesteryl esters (CEs) delivered by the endocytosed LDL are hydrolyzed by lysosomal acid lipase in the lysosomes. The released unesterified (free) cholesterol is transported to the endoplasmic reticulum (ER), in which the cholesterol is re-esterified to form CEs. The CEs are stored in cytoplasmic lipid droplets (LDs) or are transported to the cell membrane or mitochondria. HDL binds to scavenger receptor class B type 1 (SR-B1) on the cell surface, and CEs are selectively transported into the cells without the internalization of the whole lipoprotein molecule. Subsequently, the CEs are hydrolyzed into free cholesterol by hormone-sensitive lipase (HSL). This mechanism is used by steroidogenic cells that rely on cholesterol as a precursor. Large amounts of CE-rich LDs are found in steroidogenic cells, in which the CEs are hydrolyzed into cholesterol by HSL to supply the steroidogenic cells [46]. Each has a unique receptor specificity and mechanism of transporting cholesterol. SR-B1 is a lipoprotein receptor [47] and plays an important role in cholesterol efflux and steroid hormone production [48]. In addition, SR-B1 has an RCT function and determines HDL-C levels through selectively transporting HDL-CEs to the liver, where the HDL-CEs are converted to bile acids, or through biliary cholesterol secretion [49]. Various mouse model studies have shown that SR-B1-null mice exhibit an increase in the amount and size of HDL-CE, which is accompanied by a reduced level of cholesterol in secreted bile and accelerated atherosclerosis. In contrast, SR-B1-overexpressing mice exhibit decreased levels of HDL-CE, a reduced degree of atherosclerosis, and a reduced extent of atherosclerosis in the liver [49]. Binding of HDL to SR-B1 increases anti-inflammatory cytokines, such as IL-10 and transforming growth factor-beta (TGF-β), by initiating Akt and reducing NF-κB activation, thereby regulating the inflammatory response of macrophages. SR-B1 in macrophages also regulates efferocytosis or the removal of apoptotic cells through the Src/phosphoinositide 3-kinase (PI3K)/Akt/Rac1 signaling pathway, thereby enhancing survival and the anti-inflammatory response of phagocytes. In endothelial cells, SR-B1 participates in the translocation of HDL from the apical to basolateral side, which further promotes the removal of cholesterol from intimal macrophages and lymphatic vessels [50].

### 3.2. The Correlation Between Psoriasis and Atherosclerosis

Patients with psoriasis are at a high risk for the incidence of cardiovascular disease and myocardial infarction [51]. Compared with the general population, the risk factors for cardiovascular diseases in patients with psoriasis have a high incidence, such as diabetes, hypertension, obesity, and hyperlipidemia [51]. Psoriasis is highly correlated with cardiovascular diseases, and hyperlipidemia-induced atherosclerosis is the predominant disease [1]. A large amount of evidence indicates that psoriasis is related to severe atherosclerosis and cardiovascular diseases while inflammation plays an important role in psoriasis and atherosclerosis [1]. Atherosclerosis and psoriasis have very similar immune response profiles. In addition, atherosclerosis and psoriasis share the same immunological pathways (including DCs, Th1 cells, Th17 cells, and Tregs) as well as angiogenic factors and oxidative mechanisms [2]. A number of studies show that patients with psoriasis exhibit decreased HDL levels and/or increased low-density lipoprotein (LDL), very-low-density lipoprotein (VLDL), and triglyceride (TG) levels [3].

### 3.3. The Relationship Between Psoriasis and Blood Lipids

Obesity is related to many immune diseases, including cardiovascular diseases, arteriosclerosis, and psoriasis [52]. However, in mouse models, both obese and lean mice develop severe psoriasiform skin inflammation after being fed a high-fat diet (HFD). Such a phenomenon is related to FFAs but is not related to the fat mass extension, blood glucose levels, and adipose tissue-derived mediators. The increase in FFAs leads to an immune response and insulin resistance, which alters the homeostasis and function of immune cells. HFD also increases the level of saturated fatty acids (SFAs). SFAs stimulate myeloid cells to produce TLR-like stimuli, which induce a pro-immune response, thus resulting in keratinocyte activation. Therefore, decreasing the SFA content in food reduces psoriasiform inflammation. HFD-induced obesity does not directly alter the proinflammatory status of skin and immune cells. However, it renders the skin and immune cells more susceptible to proinflammatory stimuli, such as imiquimod (IMQ), thereby enhancing the immune response [53]. Certain studies failed to establish a correlation between psoriasis and lipid serum levels [54], including a large population-based cross-sectional study conducted in the UK [55].

### 3.4. The Relationships among Oxidized LDL (oxLDL), the Inflammatory Response, and Psoriasis

The accumulation of excessive cholesterol in blood vessel walls could promote the dysfunction and activation of epidermal cells, which induces an inflammatory response and eventually leads to the production of pro-inflammatory cytokines and reactive oxygen species, the overexpression of adhesion molecules and chemokines, and a reduction in nitric oxide levels [4]. The above processes lead to the aggregation and invasion of monocytes and the differentiation of monocytes into macrophages. After receiving modified LDL via scavenger receptors, macrophages are converted to foam cells. In addition, TNFα triggers endothelial dysfunction and induces an inflammatory cascade in the blood vessel wall, ultimately resulting in vascular sclerosis [5]. Although elevated TNFα exerts a protective effect during acute immunity, the maintenance of a high concentration of TNFα during chronic inflammation may alter lipid and carbohydrate metabolism. In fact, TNFα decreases the concentration of LDL-C through diminishing the secretion of apolipoproteins and reducing cholesterol catabolism and excretion, thereby interfering with cholesterol metabolism. In addition, TNFα promotes the production of pro-atherogenic small dense LDL and oxLDL to alter the quality of lipoproteins. TNFα also reduces HDL-C levels and changes HDL composition [6]. Patients with chronic inflammatory responses experience qualitative and quantitative changes in lipid and lipoprotein profiles, including decreases in cholesterol, HDL-C, and apolipoprotein A-1, and increases in small dense LDL, lipoprotein(a) [Lp(a)], and TG. In addition to TNFα, IL-6 and IL-1ß also alter lipid metabolism, including increasing VLDL and reducing TG-rich lipoprotein clearance. An increase in serum TG levels enhances the expression of cholesteryl ester transfer protein (CETP), which transfers TG from VLDL/LDL to LDL/HDL. These TG-enriched particles become the substrate for hepatic lipase and lipoprotein lipase, resulting in the production of small dense LDL and HDL as the final products. Small dense LDL more readily enters into the arteries [9] and is more susceptible to oxidation. As a result, the antioxidant and anti-inflammatory activities of small dense HDL become limited [56]. LDL levels and composition are also altered due to inflammation. First, the increase in LDLR expression reduces LDL-C levels and thus promotes the intracellular accumulation of cholesterol. Second, circulating LDL is more prone to oxidation. Therefore, patients with chronic inflammatory diseases have high plasma levels of oxLDL. oxLDL is more atherogenic and can enhance the inflammatory response. Moreover, oxLDL promotes the accumulation of cholesterol and the formation of cholesterol crystals in lysosomes, which causes lysosomal disruption and leads to increased cellular toxicity [7].

### 3.5. The Relationships among HDL, the Inflammatory Response, and Psoriasis

HDL has RCT, anti-inflammatory, and antioxidant functions, which are related to the prevention of cardiovascular diseases [57]. In addition, HDL has anti-apoptotic and anti-thrombotic activities. The protective effect of HDL is mostly due to its role in RCT. RCT refers to the process of cholesterol transport from the peripheral tissues to the liver, where the cholesterol is secreted, converted into bile acid, or synthesized into sterol hormones [58]. The first step in RCT is the removal of excess cholesterol from macrophage foam cells at sclerotic sites via HDL or macrophage-derived apolipoproteins [59]. Cholesterol efflux can be achieved through a number of ways, including (a) unidirectional efflux to lipid-free apolipoproteins, particularly apoA-1, mediated by ATP-binding cassette A1 (ABCA1); (b) unidirectional efflux to mature HDL particles mediated by ABCG1; and (c) efflux to mature HDL particles via passive diffusion facilitated by SR-B1 [60]. Once cholesterol is transferred to the cytoplasm, SR-B1 delivers the macrophage-derived (HDL) cholesterol to the liver for bile acid production. The next step is biliary secretion or transport to steroidogenic tissues, especially the adrenal gland and ovary, for steroid production and storage. HDL terminates lipid peroxidation chain reactions via apolipoproteins (such as apoA) and enzymes (paraoxonase (PON1), lecithin-cholesterol acyltransferase (LCAT), and platelet activating factor-acetyl hydrolase), thereby exhibiting an antioxidant function [61]. HDL not only inhibits monocyte transmigration and the expression of adhesion molecules in endothelial cells but also plays an immunomodulating role in the innate and acquired immune systems. HDL regulates DCs, monocytes, macrophages, T cells, and B cells mainly through modification of the cholesterol content of lipid rafts [62]. The anti-inflammatory properties of HDL are achieved through apoA-1, a major HDL-associated protein. apoA-1 stimulates the production of IL-10 and prostaglandin E2 (PGE2), thereby inhibiting the differentiation and functions of DCs [63], and reducing T cell activation and IL-12 production [8]. During a chronic inflammatory response, the antioxidative and anti-inflammatory properties of HDL are reduced. In addition, RCT and the ability to resist LDL oxidation are affected. The decrease in the antioxidative property is due to proteome alterations, including decreased activity of HDL-associated enzymes and the accumulation of complement (C3), ceruloplasmin, and serum amyloid A (SAA) [58]. The reduced anti-inflammatory property may be related to decreased apoA-1 and apoM levels. In addition, enhancement of the pro-inflammatory mechanism is due to the impaired cellular efflux of lipids to HDL, which initiates intracellular STAT3 signaling and induces vascular inflammation. In addition to low plasma levels of HDL [9], HDL proteomic and lipid composition are altered in psoriasis [15,16], which leads to a decrease in the cholesterol efflux capacity [12,15] and a reduction in the anti-inflammatory and antioxidant capacities of HDL. Moreover, other properties of HDL are also altered, such as the anti-LDL oxidation capability, inhibition of TNF-α-induced monocyte adhesion to epidermal cells, prevention of the ox-LDL-induced monocyte migration, and protection of epidermal cells from TNF-α-induced apoptosis [14]. Patients with psoriasis exhibit low PON-1 activity, which is negatively correlated with psoriasis area and severity index (PASI) scores [58]. Conversely, anti-psoriatic therapy restores the composition and function of HDL [64]. HDL-associated proteins also undergo changes, among which apoA-1 and apoM are significantly reduced. In contrast, the levels of acute-phase proteins, such as SAA, prothrombin, α-1-antitrypsin, and α-1-acid glycoprotein 1, are significantly increased. The cholesterol efflux capacity is related to decreased apoA-1, phosphatidylcholine, and sphingomyelin in HDL. Under chronic inflammatory conditions, such as psoriasis, the changes in protein structure and the appearance of neo-epitopes may lead to autoantibody production and HDL dysfunction and are even related to cardiovascular diseases [10]. Therefore, these antibodies may serve as new biomarkers for autoimmune diseases in cardiovascular diseases. High titers of autoantibodies against HDL (aHDL) and apoA-I are present in chronic inflammatory diseases, which is related to high cardiovascular risk. aHDL and aapoA-I are also found in patients with psoriasis. These antibodies are correlated with the severity of diseases and may be related to the development of vascular sclerosis.

## 4. Conclusions

**Figure 1:**
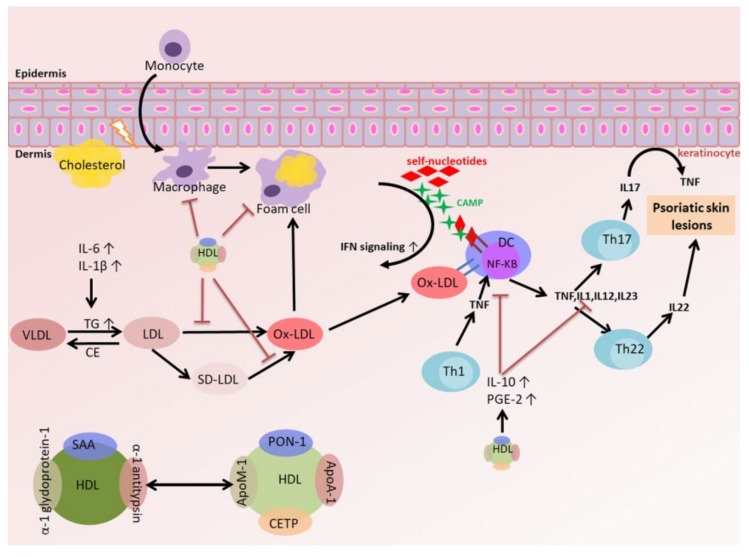
Immune cells and lipoprotein-associated cytokines implicated in psoriasis pathogenesis. Characteristic markers and cytokines related to the interleukin (IL)-17/IL-23 immune signature of T cells, lipoprotein, dendritic cells (DCs), and associated immune cells in psoriatic skin inflammation.

Psoriasis affects at least 125 million people across the world. Additional development and evaluation of the relationship between the lipid profile and psoriasis to explore treatment efficacy is required. In chronic inflammation, TNFα may influence the lipid profile, such as LDL-C levels, via decreased concentrations of apolipoproteins. Moreover, TNFα lowers the quality of lipoprotein by inducing the production of LDL and oxLDL but reducing the level of HDL-C at the same time. Oxidized LDL (oxLDL) not only exacerbates inflammation but also promotes cholesterol accumulation in lysosomes, which eventually leads to cell death. On the other hand, HDL has a reverse cholesterol transport (RCT) function, anti-oxidative capacity, and anti-inflammatory properties by regulating dendritic cells’ (DCs) differentiation and reducing T cell activation and cytokine production. However, these properties are reduced during chronic inflammation, such as psoriasis. Thus, identification of the relationship between hyperlipidemia and psoriasis is of paramount importance to develop a new therapeutic prospect for psoriasis (Figure 1).

## Abbreviations

apoA-I	Apolipoprotein A1
ABCA1	ATP-binding cassette A1
aHDL	autoantibodies against HDL
AMP	Antimicrobial peptide
apo	apolipoprotein
BMI	Body mass index
C3	Complement 3
CAMP	Cathelicidin antimicrobial peptide
CCL20	C-C motif chemokine ligand 20
CD	Cluster of differentiation
CE	Cholesteryl ester
CER	Ceramide
CETP	Cholesteryl ester transfer protein
DC	Dendritic cell
ER	Endoplasmic reticulum
FFA	Free fatty acid
GM-CSF	Granulocyte macrophage colony stimulating factor
HDL	High density lipoprotein
HDL-C	High-density lipoprotein cholesterol
HDL-CE	High density lipoprotein cholesteryl ester
HFD	High fat diet
HSL	Hormone-sensitive lipase
ICAM-1	Intercellular Adhesion Molecule 1
IFN	Interferon
IL	Interleukin
IMQ	Imiquimod
JAK	Janus kinase
LCAT	Lecithin-cholesterol acyltransferase
LD	Lipid droplet
LDL	Low density lipoprotein
LDL-R	Low density lipoprotein receptor
Lp(a)	Lipoprotein(a)
MCP-1	Monocyte chemoattractant protein 1
MHC	Major histocompatibility complex
NF-κB	Nuclear factor-κB
NK	Natural killer
ox-LDL	Oxidized LDL
pDC	Plasmacytoid dendritic cell
PI3K	Phosphoinositide 3 kinase
PON	Paraoxonase
PsA	Psoriatic arthritis
PV	Psoriasis vulgaris
RCT	Reverse cholesterol transport
SAA	Serum amyloid A
SFA	Saturated fatty acid
SR-B1	Scavenger receptor class B type 1
STAT	Signal transducer and activator of transcription
Th	T helper cell
Tfh	T follicular helper cells
TG	Triglyceride
TGF-β	Transforming growth factor beta
TLR	Toll-like receptor
TNF	Tumor necrosis factor
Treg	Regulatory T cell
VCAM-1	Vascular cell adhesion molecule 1
VLDL	Very-low-density lipoprotein
